# A rare disorder or not? How a child with jaundice changed a nationwide regimen in the Netherlands

**DOI:** 10.1007/s12687-017-0330-8

**Published:** 2017-09-15

**Authors:** E. A. L. van den Heuvel, A. Baauw, S. J. Mensink-Dillingh, M. Bartels

**Affiliations:** 1 Department of Pediatrics, Gelderse Vallei Hospital, Ede, the Netherlands; 2 Department of Pediatric Hematology and Stem Cell Transplantation, University Medical Center Utrecht, Lundlaan 6, 3508 AB Utrecht, the Netherlands; 3 Department of Pediatrics, Rijnstate Hospital, Arnhem, the Netherlands; 4 Taskforce Refugee and Migrant Children; Committee International Child Health, Dutch Society of Pediatrics, Utrecht, the Netherlands

## Abstract

Due to global migration, there is an increased frequency of diseases, which used to be rare in Western countries. Here, we describe a striking case in order to create awareness for diseases that are known for decades but sometimes “forgotten” in Western countries, including glucose-6-phosphate dehydrogenase deficiency. We will discuss how everyday practice can lead to serious medical problems and present general recommendations to support.

**Electronic supplementary material:**

The online version of this article (10.1007/s12687-017-0330-8) contains supplementary material, which is available to authorized users.

## Introduction

Glucose-6-phosphate dehydrogenase (G6PD) deficiency is the most prevalent red blood cell enzyme deficiency in the world, affecting more than 400 million people worldwide, and one of the best characterized genetic disorders (Nkhoma et al. 2009). It is a hereditary X-linked disorder, based on mutations in the *G6PD* gene that manifests itself in a spectrum of non-immune-mediated hemolytic anemias. Most patients with G6PD deficiency are asymptomatic and do not suffer from hemolysis in the steady state. However, episodes of acute hemolysis with hemolytic anemia may be triggered by medication, certain foods and especially infections (Cappellini and Fiorelli 2008). In neonates, severe hemolytic episodes can occur resulting in extreme hyperbilirubinemia and kernicterus. Neonatal screening programs for the condition are available but are not performed worldwide (Kaplan and Hammerman 2011). Recognizing and managing chronic disorders, including G6PD deficiency by people outside the well-organized healthcare system, is an important health care issue. Here, we present a clinical case, illustrating the difficulties of preventive care for a well-known, yet often not considered disorder, in order to create awareness among doctors, and other healthcare workers involved in both the general practice, and more specialized health care institutions.

## Case

Patient A, a 9-year-old boy, was sent to the emergency department of our hospital because of fever, jaundice, and dark urine. On the day of presentation, he appeared increasingly icteric and had a mild fever. He is a Syrian refugee and resided with his aunt and two nieces in the nearby asylum seekers center. Since his biological mother was still staying in Syria and his biological father had died, his medical history was largely unknown. Physical examination showed a moderately ill boy, who was very icteric with pale mucous membranes. His blood oxygen level was 81%, which did not improve with 100% oxygen and he had a tachycardia. His urine appeared black and foamy; further physical examination showed no abnormalities. Additional laboratory tests were performed, yet the results were difficult to interpret as a result of severe hemolysis. His hemoglobin (Hb) was 4 mmol/L (normal 7.4–9.0 mmol/L), reticulocytes were 21 × 10^9^/L (normal 25–120 × 10^9^/L), platelets were 288 × 10^9^/L (normal 150–450 × 10^9^/L), indirect bilirubin was 90 μmol/L (normal 3–21 μmol/L), haptoglobin was 0.16 g/L (normal 0.3–2 g/L), and direct antiglobuline test (DAT/Coombs) was negative. A stained blood smear showed no malaria parasites. Based on these results, we diagnosed an acute hemolytic anemia, which was most likely not immune-mediated (DAT negative).

Upon further inquiry, he was found to have eaten fava beans 3 days before, suggesting an underlying glucose-6-phosphate dehydrogenase (G6PD) deficiency as the cause of this hemolytic crisis. He was admitted to the pediatric department for further analysis and treatment. He was treated with a red cell transfusion, leading to both clinical and hematological improvement. Analysis of G6PD levels demonstrated a markedly reduced glucose-6P-dehydrogenase enzyme activity in erythrocytes (0.1 IU/g Hb, normal 3.8–5.9 IU/g Hb) in fluorescent spot test.

Since G6PD deficiency is a hereditary disorder, we asked about the welfare of the patient’s family members. Patient B, a 6-year-old girl (niece of patient A), was becoming increasingly icteric and also produced dark urine, after having the same fava-bean dinner. She had no physical complaints and had always been healthy. Physical examination showed a mildly icteric girl with normal vital parameters, who also produced dark urine. Further physical examination showed no abnormalities. She had a mild hemolysis, most likely due to G6PD deficiency. Diagnostic tests were performed, followed by clinical observation and no therapeutic interventions. She turned out to be a heterozygous carrier of G6PD deficiency based on a reduced glucose-6P-dehydrogenase enzyme activity in erythrocytes (2.7 IU/g Hb, normal 3.8–5.9 IU/g Hb) in fluorescent spot test.

## Pathophysiology

**Fig. 1:**
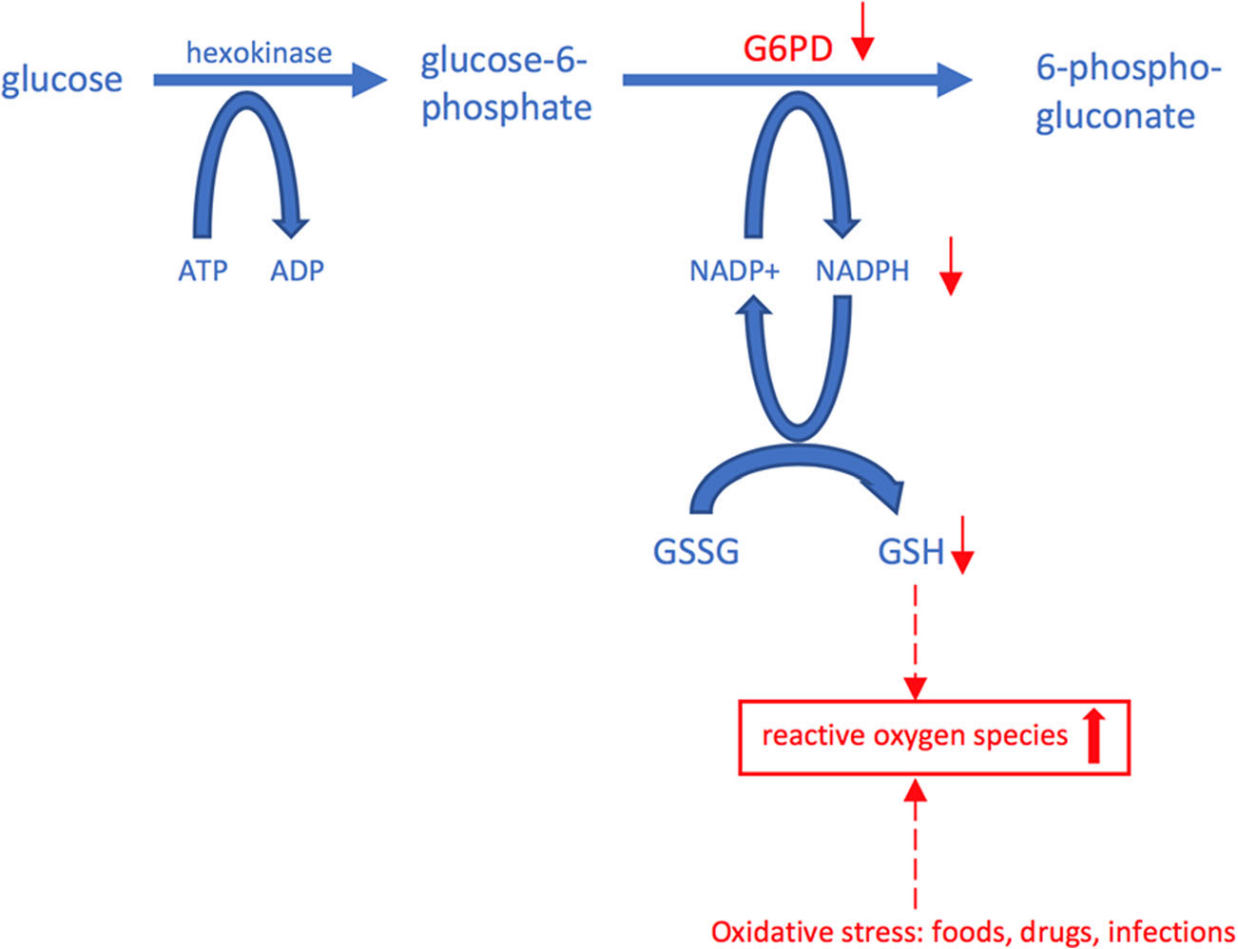
The role of G6PD in the pentose phosphate pathway

Glucose-6-phosphate dehydrogenase plays an essential role in the glutathione system that protects cells from the harmful effect of oxygen radicals. Reduced glutathione can neutralize oxygen radicals into water and is oxidized in this process itself. For this reaction, oxidized nicotinamide adenine dinucleotide phosphate (NADPH) is required. Glucose-6-phosphate dehydrogenase converts NADP to NADPH and is the only source for this reaction in the red blood cell (RBC). Without glucose-6-phosphate-dehydrogenase, there is a deficiency of reduced glutathione, and the erythrocyte is not protected against oxygen radicals (Fig. 1.)

Most tissues compensate for reduced G6PD activity by increasing G6PD protein synthesis, thereby increasing residual activity. Since erythrocytes have no nucleus, and as a consequence no intrinsic protein synthesis, erythrocytes are more sensitive to oxidative stress compared to other cell types. In case of increased oxidative stress, for instance during infections, this induces acute hemolysis. Recovery from acute hemolysis can occur spontaneously as the triggering factor disappears, and compensatory reticulocytosis takes place. Compared to erythrocytes, reticulocytes have a relatively high G6PD activity.

More than 140 mutations in the *G6PD* gene, located on X-chromosome (Xq28), have been described so far. The World Health Organization (WHO) has made a classification of the different G6PD deficiency variants, based on the severity of hemolysis (Table 1).

**Table 1:** Classification of G6PD deficiency

WHO	Synonyms	Severity of hemolysis	Enzyme activity	Clinical manifestations
Class 1		Chronic hemolysis	Severe deficiency (< 10% of normal)	Congenital hemolysis (rare)
Class 2	G6PD Mediterranean type	Intermitting severe hemolysis after exposure to oxidant stress	Severe deficiency (< 10% of normal)	Most common class
Class 3	G6PD A-	Intermitting hemolysis after exposure to oxidant stress	10–60% of normal	Mild phenotype
Class 4/5	G6DP A+/ G6PD B	No hemolysis	Normal or decreased	Not clinically relevant

Some patients with severe G6PD deficiency suffer from chronic hemolysis; however, this is rare. Chronical hemolysis due to G6PD deficiency variants leads to moderately severe chronic hemolysis, which increases by oxidative stress. This group is named chronic non-spherocytic hemolytic anemias (CNSHA) and is classified as group 1 G6PD deficiency by the WHO. The G6PD variants causing CNSHA all arise from independent mutations of which 61 molecular variants have been identified (Fiorelli et al. 2000). Life expectancy and quality of life are generally not affected by G6PD deficiency (Cocco et al. 1998).

While male patients suffer from a functional deficiency, female carriers usually do not suffer from severe hemolysis, since half of the red blood cells express the normal G6PD allele. Still, G6PD deficiency can be clinically relevant in females, due to unfavorable lyonization or as a result of the inheritance of two affected alleles (homozygosity or compound heterozygosity), although this is rare. Therefore, it is very important to gain full family history, in particular concerning male family members.

The severity of hemolysis varies among individuals and therefore the clinical manifestations encompass a spectrum. Most children with G6PD deficiency will have no problems in daily life. The illness manifests as oxidative stress is triggered. These triggers can be provoked by roughly three types of oxidative stress as follows: (1) infections, in particular hepatitis A and B virus infections (uncommon in the Netherlands), pneumonia, cytomegalovirus (CMV) infection, and typhoid fever (Cappellini and Fiorelli 2008); (2) medication, leading to drug-induced hemolytic anemia (listed in Electronic Supplementary Material Table 1 Youngster et al. 2010); and (3) favism, which has been reported since the beginning of the twentieth century and is mainly induced by eating fresh fava beans, but can occur after eating dried or frozen beans. Favism was originally noted in Mediterranean countries, but with the growing consumption of fava beans due to worldwide distribution, it is also common in the Middle East and North Africa (Belsey 1973).

### Epidemiology

G6PD deficiency is global in its distribution (Cappellini and Fiorelli 2008) and occurs especially in tropical and subtropical areas of the Eastern hemisphere, parallel to the malaria distribution. A conservative estimate is that 400 million people worldwide carry the *G6DP* gene mutation. The highest prevalence is measured in countries like Namibia, Angola, Congo-Kinshasa, Egypt, and Saudi Arabia where 15–26% of the population carries a mutation. In Libya, Syria, Iraq, Sudan, and Afghanistan, 7–10% of the population is carrier (Cappellini and Fiorelli 2008). The prevalence of G6PD deficiency in the Netherlands is around 0.1%. Due to immigration, G6DP deficiency is now found with increasing frequency in non-endemic areas. The prevalence in which this occurs is unknown. A recent study in Denmark demonstrated a G6PD deficiency frequency of 2.9% in female immigrants and 12% in male immigrants from a selection of 1500 random immigrants who had an indication for hematological testing (Warny et al. 2015).

### Diagnostic evaluation, management, and neonatal screening

The most effective management strategy for G6PD deficiency is to prevent hemolysis by avoiding oxidative stressors, see Electronic Supplementary Material (ESM) for an extensive list. This requires awareness of the diagnosis among the patient and caregivers and treating healthcare workers.

Whenever mild hemolysis occurs in individuals with G6PD deficiency, the most important intervention is to remove the inciting agent(s). In the majority of cases, additional interventions are not needed. In case of severe hemolysis, acute management should be focused on generally stabilizing the patient according to Advance Pediatric Life Support (APLS) guidelines, including erythrocyte transfusions combined with adequate hydration if necessary. If recurrent transfusions of RBC’s are needed in patients with G6PD deficiency/CNSHA, there is a clinically significant risk of iron overload and ferritin levels should be monitored. In patients suffering from chronic hemolysis (CNSHA), requiring a high rate of compensatory erythropoiesis, supplementary folic acid is prescribed. There is no supportive evidence for administration of antioxidants such as vitamin E in acute hemolysis (Cappellini and Fiorelli 2008).

When a patient is suspected to suffer from G6DP deficiency, it is essential to conduct hematological testing for diagnosis prior to an erythrocyte transfusion. Diagnostic evaluation includes measuring G6PD enzyme activity by spectrophotometric analysis of metabolites of G6PD. Importantly, since enzyme activity is tested in erythrocytes, in a setting of severe hemolysis, diagnostic testing can be complicated when erythrocytes with low enzymatic activity have been removed. Therefore, in clinically severe forms of G6PD deficiency, genetic testing of the underlying genetic defect is performed to further complete the diagnostic evaluation.

There is no curative therapy for G6PD deficiency. Therefore, prevention and awareness are crucial. The WHO recommended population screening for all newborn babies in areas with prevalence of 3–5% or more in males in association with an educational campaign for mothers and health care workers, almost 30 years ago (WHO workgroup 1989). A recently performed study showed a positive impact of these screening programs combined with parental input (Kaplan et al. 2015).

## Discussion

G6PD deficiency is a chronic and potentially life-threatening disorder when patients and risk factors for acute hemolytic crises are not identified adequately. As a consequence of extensive global migration, it is important to realize that there will be significantly more (asymptomatic) patients who will need medical care within healthcare systems, which leads to a diagnostic challenge. In 2015, 43,093 refugees applied for asylum in the Netherlands and another 13,854 were reunited with a family member who were already staying here. The majority of these refugees are Syrian (Online document Tjin-A-Tsoi TBPM, Jaarrapport integratie 2016). By estimation, between 1651 and 4840 carriers of a *G6PD* gene mutation (prevalence 2.9–8.5%) settled in the Netherlands in 2015.

Global migration leads to a growing ethnically heterogeneous population in which the exact prevalence of G6PD deficiency is largely unknown. In order to provide adequate health care for this group according to Dutch health care standards, it is important to increase our knowledge about both the epidemiologic and the pathophysiological features of G6PD deficiency and other relatively rare disorders that were relatively rare in the Dutch population until recently.

Concerning G6PD deficiency, caregivers, healthcare workers, and national health authorities must be trained to recognize disease symptoms and potential risk factors that might trigger complications of G6PD deficiency. As an example, something trivial as the menu (fava beans) in an asylums seekers center can have major consequences, which is illustrated by our case story. Furthermore, it needs to be considered whether in addition to standard screening for infectious diseases (e.g., TBC, hepatitis), immigrants should be screened for G6PD deficiency.

Following the presentation of our case, the Dutch Association of Pediatrics published a press release in which they advised to stop serving fava beans in asylum seeker centers and thereby avoid unnecessary health risks. While the relation between favism and the risk of hemolytic crisis in G6PD deficiency has been known for centuries, incidents similar to our case still occur relatively frequent (Cappellini and Fiorelli 2008). Clearly, it is difficult to recognize a disease with such a genetic diversity and broad clinical spectrum. Recognizing and/or identifying high-risk patients and managing G6PD deficiency in immigrants is therefore an important health care issue.

The WHO recommends no obligatory screening of refugee and migrant populations for diseases in general, because there is no clear evidence of benefits (or cost-effectiveness). WHO strongly recommends, however, that health checks be offered and provided to ensure access to health care for all refugees and migrants requiring health protection (Migration and health: key issues, WHO 2017). Screening for G6PD deficiency in migrants living in low-G6PD deficiency frequency areas is contradictory in recent literature. There is no justification for screening based on the low frequency of G6PD deficiency (2.9%) found in groups migrating to Denmark (Warny et al. 2015). Other studies suggest that it can be beneficial since severe phenotypes may be more frequent in countries of origin in this group of people and they will benefit from (neonatal) screening (WHO workgroup 1989).

When asylum seekers enter the Netherlands, they receive an invite for medical screening within a few days. The intention of this screening is to gain insight in the health situation and search for signals with regard to health risks (physical, psychological, psychosocial). The intake is divided into two moments of contact: information collected by a nurse and a doctor’s examination. The nurse obtains the medical history and lists current problems. The pediatrician completes the medical information, performs a general physical examination, and establishes a (catch up) vaccination plan followed by a screening for active tuberculosis. There is no standard questionnaire for doctors/nurses and therefore it is possible that full and relevant medical history is not obtained. It should be considered to set up a more elaborate screening program in which not only G6DP deficiency can be included but also other diseases like thalassemia and severe infections. Preferably, the extent of this screening program should be discussed on a nationwide public healthcare level.

Concerning G6PD deficiency, ideally pharmacogenetic tests will be performed in patients when they are diagnosed. Pharmacogenetic tests (PGx) are developed to identify an individual’s presence, absence, or genetic variant that influence drug response (Kalman et al. 2016). The results of these tests can help healthcare providers to select the most effective drugs and dose for patient and can identify potential toxic drugs for the individual. In 2015, about 150 different drugs that are approved by the US Food and Drug Administration (FDA) include pharmacogentic information on the label, and only a few of them have recommendations for PGx testing. Similar results are found in medication approved by the European Medicine Agency (Wietzel et al., 2017). Suggestions for standardized PGx test panels are made in literature but testing for G6DP deficiency is not mentioned in these articles. In addition, studies are mostly performed on genetic profiles of Western patients and therefore the relevance of G6PD deficiency and the severe (and potential life-threatening) complication seems to be “forgotten” in Western countries. Although technically within reach (Luzzato and Seneca 2014), at this point it is not included in the standard care for G6PD-deficient patients. In the near future, this can help doctors distinguish which drugs are safe for G6PD-deficient patients individually.

## Conclusion

The sizeable global migration forces doctors in Western countries to look critically at the specific medical care provided to groups of immigrants within the public healthcare services. There is an increased frequency of diseases and complications, which used to be rare in Western countries. Awareness of ensuing problems among doctors and health care workers needs to be improved, which represents a challenge for national health authorities. We recommend development of a standard questionnaire and suitable screening program to assemble full medical data, which then can be used to determine prevalence of specified diseases and resulting therefrom in improved healthcare for immigrants and their children.

## Electronic supplementary material

Table 1 (DOCX 62 kb)

Appendix 1 (DOCX 14 kb)

## References

[CR1] Belsey M (1973). The epidemiology of favism. Bull World Health Organ.

[CR2] Cappellini MD, Fiorelli G (2008). Glucose-6-phosphate dehydrogenase deficiency. Lancet.

[CR3] Cocco P, Todde P, Fornera S (1998). Mortality in a cohort of men expressing the glucose-6-phosphate dehydrogenase deficiency. Blood.

[CR4] Fiorelli G, Martinez di Montemuros F, Cappellini MD (2000) Chronic non-spherocytic haemolytic disorders associated with glucose-6-phosphate dehydrogenase variants. Balliere Clinical Haemotology 39–55 10.1053/beha.1999.0056 10916677

[CR5] Kalman LV, Agundez JAG, Lindqvist Appell M (2016). Pharmacogenetic allele nomenclature; international workgroup recommendations for test result reporting. Clin Pharmacol Therp, feb.

[CR6] Kaplan M, Hammerman C (2011). Neonatal screening for glucose- 6-phosphate dehydrogenase deficiency: biochemical versus genetic technologies. Semin Perinatol.

[CR7] Kaplan M, Hammerman C, Bhutanu VK (2015). Parental education and the WHO neonatal G-6-PD screening program; a quarter century later. J Perinatol.

[CR8] Luzzato L, Seneca E (2014). G6DP deficiency; a classic example of pharmacogenetics with on-going clinical implications. Br J Haematol.

[CR9] Migration and health: key issues, WHO 2017 http://www.euro.who.int/en/health-topics/health-determinants/migration-and-health/migrant-health-in-the-european-region/migration-and-health-key-issues#292934

[CR10] Nkhoma ET, Poole C, Vannappagari Vm Hall SA, Beutler E (2009). The global prevalence of glucose-6-phophate dehydrogenase deficiency; a systematic review and meta-analysis. Blood Cells Mol Dis.

[CR11] Online document Tjin-A-Tsoi TBPM, Jaarrapport integratie (2016) Centraal Bureau voor de statistiek. https://wwwcbsnl/nl-nl/publicatie/2016/47/jaarrapport-integratie-2016 (Nov 2016)

[CR12] Warny M, Wirenfelft Klausen T, Petersen BH (2015). Prevalence of glucose-6-phospate dehydrogenase deficiency and diagnostic challenges in 1500 immingrants in Denmark examined for haemoglobinopathies. Scandinavian journal of clinical & laboratory investigations.

[CR13] WHO workgroup (1989). Glucose-6-phosphate dehydrogenase deficiency. Bull World Health Organ.

[CR14] Wietzel KW, Cavallari LH, Lesko LJ (2017) Preemptive panel-based pharmacogentic testing: the time is now. Pharm Res. 10.1007/s11095-017-2163-x 10.1007/s11095-017-2163-x PMC5518315 28466392

[CR15] Youngster I, Arcavi L, Schechmaster R (2010). Medications and glucose6-phosphate dehydrogenase deficiency. Drug Saf 2010.

