# Supplementary material for: A rare disorder or not? How a child with jaundice changed a nationwide regimen in the Netherlands
Source: J Community Genet. 2017 Sep 15;8(4):335–9. doi: 10.1007/s12687-017-0330-8 (PMC5614890; doi:10.1007/s12687-017-0330-8)
Supplement: Supplementary file 1 — (DOCX 62 kb) [file 12687_2017_330_MOESM1_ESM.docx]

| References 1,15 | Unsafe | Previously unsafe, but probably safe in therapeutic dose |
| --- | --- | --- |
| Anti-infective | Dapsone  Nitrofurantione (and related)  Primaquine  Pamaquine  Sulfanilamide  Sulfacetamide  Sulfapyridine  Sulfamethoxazole | Clorquine  Mepacrine  Quinine  Sulfadimidine  Sulfasalazine  Aldesulfone  Glibenclamide  Co-trimoxazol  Ciprofloxacin  Levofloxacin  Nalidic acid  Norfloxacin  Ofloxain |
| Miscellaneous | Acetanilide  Uricase (rasburicase, pegloticase)  Phenazopyridine  Methylthioninium chloride (methylene blue)  Dimercaprol  Nalidixic acid  Niridazole  Methylthionium | Asprin  Acetaminophen  Antipyrin  Aminophenzone (and related NSAID’s)  Ascorbic acid  Mesalazine  Vitamin K analogues  Chloramphenicol  Doxorubicin  Dimercaprol  Probenecid |
| Chemicals and foods | Fava beans  Aniline dyes  Naphthalene (mothballs, lavatory deodorant)  Henna compounds (and related dyes used for hair and tattoos)  2,4,6-trinitrotoluene | Acalypha indica extract |

Electronic Supplementary Material Table 1: Drugs and chemicals associated with substantial hemolysis in patients with G6PD deficiency
